# Supplementary material for: Changes in Sitting Time, Screen Exposure and Physical Activity during COVID-19 Lockdown in South American Adults: A Cross-Sectional Study
Source: Int J Environ Res Public Health. 2021 May 14;18(10):5239. doi: 10.3390/ijerph18105239 (PMC8156285; doi:10.3390/ijerph18105239)
Supplement: Supplementary file 1 [file ijerph-18-05239-s001.zip › ijerph-1195258-supplementary.pdf]

Table S1. Association between differences pre-during COVID-19 lockdown in physical activity, sitting time and screen exposure and sociodemographic factors.

		Argentina			Chile			Total		
		**Δ Sitting Time (min/day) β[b]**	**95% CI[a]**	***p***	**Δ Sitting Time (min/day) β[b]**	**95% CI[a]**	***p***	**Δ Sitting Time (min/day) β[b]**	**95% CI[a]**	***p***
Age Intervals*	18-24	Ref	-	-	Ref	-	-	Ref	-	-
	25-34	-0.11	-117.29 ; 20.56	0.169	-0.06	-77.10 ; 14.21	0.177	**-0.09**	**-78.99 ; -5.75**	**0.023**
	35-44	**-0.19**	**-152.32 ; -6.85**	**0.032**	**-0.29**	**-173.06 ; -75.92**	**<0.001**	**-0.27**	**-151.92 ; -74.37**	**<0.001**
	45-54	**-0.24**	**-189.54 ; -34.14**	**0.005**	**-0.16**	**-128.99 ; -23.13**	**0.005**	**-0.20**	**-137.79 ; -54.10**	**<0.001**
	55-64	**-0.21**	**-163.92 ; -11.19**	**0.025**	**-0.22**	**-165.33 ; -56.31**	**<0.001**	**-0.22**	**-145.36 ; -60.69**	**<0.001**
	>=65	**-0.25**	**-202.28 ; -39.36**	**0.004**	**-0.10**	**-148.38 ; -7.32**	**0.031**	**-0.15**	**-142.91 ; -45.23**	**<0.001**
Sex	Male	Ref	-	-	Ref	-	-	Ref	-	-
	Female	0.06	-9.87 ; 60.23	0.159	0.06	-6.24 ; 61.18	0.110	**0.06**	**2.93 ; 51.77**	**0.028**
Educational Level	Low	Ref	-	-	Ref	-	-	Ref	-	-
	Middle	-0.19	-166.57 ; 16.38	0.107	0.10	-12.04 ; 105.14	0.119	0.06	-21.13 ; 74.97	0.272
	High	-0.18	-159.14 ; 18.01	0.118	**0.15**	**9.84 ; 114.78**	**0.020**	0.09	-5.14 ; 84.63	0.083
Area of Residence	North	Ref	-	-	Ref	-	-	Ref	-	-
	Center	**-0.18**	**-151.32 ; -15.82**	**0.016**	**0.14**	**16.43 ; 136.65**	**0.013**	0.02	-33.55 ; 57.07	0.611
	South	-0.13	-149.67 ; 6.55	0.072	0.10	-3.95 ; 142.42	0.064	0.03	-34.45 ; 72.61	0.484
Marital Status	Single	Ref	-	-	Ref	-	-	Ref	-	-
	Married/ Civil Union/ Cohabiting	0.05	-22.70 ; 54.12	0.422	**-0.12**	**-79.67 ; -8.20**	**0.016**	-0.05	-44.21 ; 8.50	0.184
	Divorces/ Separated	-0.02	-59.01 ; 43.35	0.764	0.01	-40.45 ; 51.91	0.808	-0.01	-37.30 ; 31.96	0.880
	Widow	-0.03	-99.06 ; 55.69	0.582	-0.05	-211.25 ; 30.62	0.143	-0.05	-112.07 ; 15.20	0.136

		**Δ Screen Exposure (min/day) β[b]**	**95% CI[a]**	***p***	**Δ Screen Exposure (min/day) β[b]**	**95% CI[a]**	***p***	**Δ Screen Exposure (min/day) β[b]**	**95% CI[a]**	***p***
Age Intervals*	18-24	Ref	-	-	Ref	-	-	Ref	-	-
	25-34	-0.01	-76.93 ; 69.98	0.926	**-0.16**	**-132.38 ; -35.69**	**0.001**	**-0.12**	**-97.30 ; -20.09**	**0.003**
	35-44	-0.11	-127.42 ; 27.62	0.207	**-0.32**	**-194.19 ; -91.33**	**<0.001**	**-0.26**	**-158.84 ; -77.08**	**<0.001**
	45-54	-0.07	-115.35 ; 50.27	0.441	**-0.19**	**-154.47 ; -42.37**	**0.001**	**-0.17**	**-129.59 ; -41.35**	**<0.001**
	55-64	**-0.21**	**-175.42 ; -12.63**	**0.024**	**-0.26**	**-194.83 ; -79.39**	**<0.001**	**-0.27**	**-177.07 ; -87.82**	**<0.001**
	>=65	**-0.21**	**-196.81 ; -23.17**	**0.013**	**-0.11**	**-165.71 ; -16.32**	**0017**	**-0.19**	**-173.51 ; -70.52**	**<0.001**
Sex	Male	Ref	-	-	Ref	-	-	Ref	-	-
	Female	0.12	**18.75 ; 93.46**	**0.003**	0.03	-20.21 ; 51.19	0.395	**0.07**	**9.34 ; 60.84**	**0.008**
Educational Level	Low	Ref	-	-	Ref	-	-	Ref	-	-
	Middle	-0.08	-129.40 ; 65.58	0.521	-0.07	-94.56 ; 29.55	0.304	-0.05	-75.74 ; 25.58	0.332
	High	-0.03	-108.27 ; 80.54	0.773	-0.03	-69.08 ; 42.05	0.633	-0.01	-48.20 ; 46.44	0.971
Area of Residence	North	Ref	-	-	Ref	-	-	Ref	-	-
	Center	-0.07	-105.67 ; 38.75	0.363	**0.16**	**25.97 ; 153.29**	**0.006**	0.08	-6.02 ; 89.51	0.087
	South	-0.02	-96.33 ; 70.17	0.758	**0.12**	**4.36 ; 159.36**	**0.038**	0.08	-7.37 ; 105.51	0.088
Marital Status	Single	Ref	-	-	Ref	-	-	Ref	-	-
	Married/ Civil Union/ Cohabiting	-0.04	-55.73 ; 26.14	0.478	**-0.10**	**-77.71 ; -2.02**	**0.039**	-0.07	-54.88 ; 0.70	0.056
	Divorces/ Separated	-0.03	-71.56 ; 37.54	0.541	-0.03	-66.70 ; 31.11	0.475	-0.04	-56.12 ; 16.90	0.292
	Widow	-0.01	-82.58 ; 82.34	0.998	-0.02	-162.62 ; 93.51	0.596	-0.01	-81.94 ; 52.24	0.664

		Δ MPA[c] (min/day) β[b]	95% CI[a]	*p*	Δ MPA[c] (min/day) β[b]	95% CI[a]	*p*	Δ MPA[c] (min/day) β[b]	95% CI[a]	*p*
Age Intervals	18-24	Ref	-	-	Ref	-	-	Ref	-	-
	25-34	0.08	-19.47 ; 57.81	0.330	-0.05	-41.11 ; 12.80	0.303	0.01	-18.63 ; 23.37	0.825
	35-44	0.10	-17.43 ; 64.13	0.261	-0.02	-23.93 ; 33.42	0.745	0.06	-7.51 ; 36.98	0.194
	45-54	0.02	-37.20 ; 49.93	0.774	-0.04	-42.20 ; 20.30	0.492	-0.01	-26.63 ; 21.37	0.830
	55-64	0.10	-18.60 ; 67.04	0.267	0.03	-22.49 ; 41.88	0.554	0.07	-4.95 ; 43.62	0.118
	>=65	0.08	-23.40 ; 67.95	0.339	0.02	-32.60 ; 50.69	0.670	0.05	-9.70 ; 46.34	0.200
Sex	Male	Ref	-	-	Ref	-	-	Ref	-	-
	Female	-0.02	-23.18 ; 16.13	0.725	-0.02	-25.97 ; 13.84	0.550	-0.01	-17.52 ; 10.49	0.623
Educational Level	Low	Ref	-	-	Ref	-	-	Ref	-	-
	Middle	-0.15	-84.90 ; 17.68	0.199	0.03	-26.21 ; 42.99	0.634	0.02	-23.16 ; 31.96	0.754
	High	-0.17	-84.45; 14.88	0.169	0.08	-12.26 ; 49.70	0.236	0.03	-18.75 ; 32.74	0.594
Area of Residence	North	Ref	-	-	Ref	-	-	Ref	-	-
	Center	0.07	--19.92 ; 56.05	0.351	**-0.18**	**-89.99 ; -19.00**	**0.003**	-0.08	-49.72 ; 2.26	0.073
	South	0.03	-34.90 ; 52.70	0.690	-0.09	-78.56 ; 7.86	0.109	-0.06	-50.10 ; 11.31	0.216
Marital Status	Single	Ref	-	-	Ref	-	-	Ref	-	-
	Married/ Civil Union/ Cohabiting	-0.06	-32.96 ; 10.11	0.298	**0.11**	**3.30 ; 45.50**	**0.024**	0.04	-6.85 ; 23.39	0.284
	Divorces/ Separated	-0.02	-34.27 ; 23.13	0.703	0.02	-21.31 ; 33.22	0.668	0.01	-17.89 ; 21.84	0.845
	Widow	-0.06	-68.09 ; 18.67	0.264	**-0.08**	**-143.32 ; -0.51**	**0.048**	-0.05	-66.62 ; 6.39	0.106

		Δ VPA[d] (min/day) β[b]	95% CI[a]	*p*	Δ VPA[d] (min/day) β[b]	95% CI[a]	*p*	Δ VPA[d] (min/day) β[b]	95% CI[a]	*p*
Age Intervals	18-24	Ref	-	-	Ref	-	-	Ref	-	-
	25-34	0.04	-17.17 ; 30.02	0.593	0.07	-5.26 ; 31.66	0.161	0.01	-3.19 ; 24.29	0.132
	35-44	0.07	-15.61 ; 34.20	0.464	0.05	-11.53 ; 27.74	0.418	0.06	-5.23 ; 23.87	0.209
	45-54	0.02	-23.05 ; 30.15	0.793	-0.06	-33.26 ; 9.53	0.277	-0.01	-19.74 ; 11.66	0.614
	55-64	0.01	-25.85 ; 26.44	0.983	-0.07	-34.94 ; 9.13	0.251	0.07	-20.83 ; 10.93	0.541
	>=65	0.05	-19.44 ; 36.34	0.552	-0.02	-34.89 ; 22.14	0.661	0.05	-13.19 ; 23.46	0.583
Sex	Male	Ref	-	-	Ref	-	-	Ref	-	-
	Female	-0.01	-12.75 ; 11.25	0.902	**0.12**	**8.06 ; 35.31**	**0.002**	**-0.01**	**1.45 ; 19.77**	**0.023**
Educational Level	Low	Ref	-	-	Ref	-	-	Ref	-	-
	Middle	-0.09	-42.94 ; 19.70	0.467	-0.03	-29.82 ; 17.56	0.612	0.02	-23.06 ; 12.99	0.584
	High	-0.13	-47.13; 13.53	0.277	0.01	-19.23 ; 23.13	0.859	0.03	-20.16 ; 13.52	0.699
Area of Residence	North	Ref	-	-	Ref	-	-	Ref	-	-
	Center	0.05	-14.87 ; 31.52	0.481	0.03	-18.87 ; 29.73	0.661	-0.08	-9.96 ; 24.03	0.417
	South	0.02	-23.59 ; 29.90	0.817	0.09	-6.00 ; 53.17	0.118	-0.06	-7.03 ; 33.14	0.202
Marital Status	Single	Ref	-	-	Ref	-	-	Ref	-	-
	Married/ Civil Union/ Cohabiting	0.04	-8.68 ; 17.62	0.505	**0.12**	**3.41 ; 32.31**	**0.015**	**0.04**	**1.60 ; 21.38**	**0.023**
	Divorces/ Separated	0.07	-6.07 ; 28.98	0.200	0.09	-0.27 ; 37.07	0.053	**0.01**	**2.46 ; 28.44**	**0.020**
	Widow	0.02	-20.84 ; 32.15	0.675	0.02	-34.51 ; 63.27	0.564	-0.05	-11.85 ; 35.90	0.323

		Δ MVPA[e] (min/day) β[b]	95% CI[a]	*p*	Δ MVPA[e] (min/day) β[b]	95% CI[a]	*p*	Δ MVPA[e] (min/day) β[b]	95% CI[a]	*p*
Age Intervals	18-24	Ref	-	-	Ref	-	-	Ref	-	-
	25-34	0.04	-17.17 ; 30.02	0.593	0.07	-5.26 ; 31.66	0.161	0.01	-3.19 ; 24.29	0.132
	35-44	0.07	-15.61 ; 34.20	0.464	0.05	-11.53 ; 27.74	0.418	0.06	-5.23 ; 23.87	0.209
	45-54	0.02	-23.05 ; 30.15	0.793	-0.06	-33.26 ; 9.53	0.277	-0.01	-19.74 ; 11.66	0.614
	55-64	0.01	-25.85 ; 26.44	0.983	-0.07	-34.94 ; 9.13	0.251	0.07	-20.83 ; 10.93	0.541
	>=65	0.05	-19.44 ; 36.34	0.552	-0.02	-34.89 ; 22.14	0.661	0.05	-13.19 ; 23.46	0.583
Sex	Male	Ref	-	-	Ref	-	-	Ref	-	-
	Female	-0.01	-12.75 ; 11.25	0.902	**0.12**	**8.06 ; 35.31**	**0.002**	**-0.01**	**1.45 ; 19.77**	**0.023**
Educational Level	Low	Ref	-	-	Ref	-	-	Ref	-	-
	Middle	-0.09	-42.94 ; 19.70	0.467	-0.03	-29.82 ; 17.56	0.612	0.02	-23.06 ; 12.99	0.584
	High	-0.13	-47.13; 13.53	0.277	0.01	-19.23 ; 23.13	0.859	0.03	-20.16 ; 13.52	0.699
Area of Residence	North	Ref	-	-	Ref	-	-	Ref	-	-
	Center	0.05	-14.87 ; 31.52	0.481	0.03	-18.87 ; 29.73	0.661	-0.08	-9.96 ; 24.03	0.417
	South	0.02	-23.59 ; 29.90	0.817	0.09	-6.00 ; 53.17	0.118	-0.06	-7.03 ; 33.14	0.202
Marital Status	Single	Ref	-	-	Ref	-	-	Ref	-	-
	Married/ Civil Union/ Cohabiting	0.04	-8.68 ; 17.62	0.505	**0.12**	**3.41 ; 32.31**	**0.015**	**0.04**	**1.60 ; 21.38**	**0.023**
	Divorces/ Separated	0.07	-6.07 ; 28.98	0.200	0.09	-0.27 ; 37.07	0.053	**0.01**	**2.46 ; 28.44**	**0.020**
	Widow	0.02	-20.84 ; 32.15	0.675	0.02	-34.51 ; 63.27	0.564	-0.05	-11.85 ; 35.90	0.323

Δ: Differences pre – during isolation.
p: pvalue
[a]= Confidence Interval
[b]= Beta Standardized Coefficient
[c]= Moderate physical activity
[d]= Vigorous physical activity
[e]= Moderate to vigorous physical activity
*ptrend < 0.001
Black text and bold face indicate statistical significance

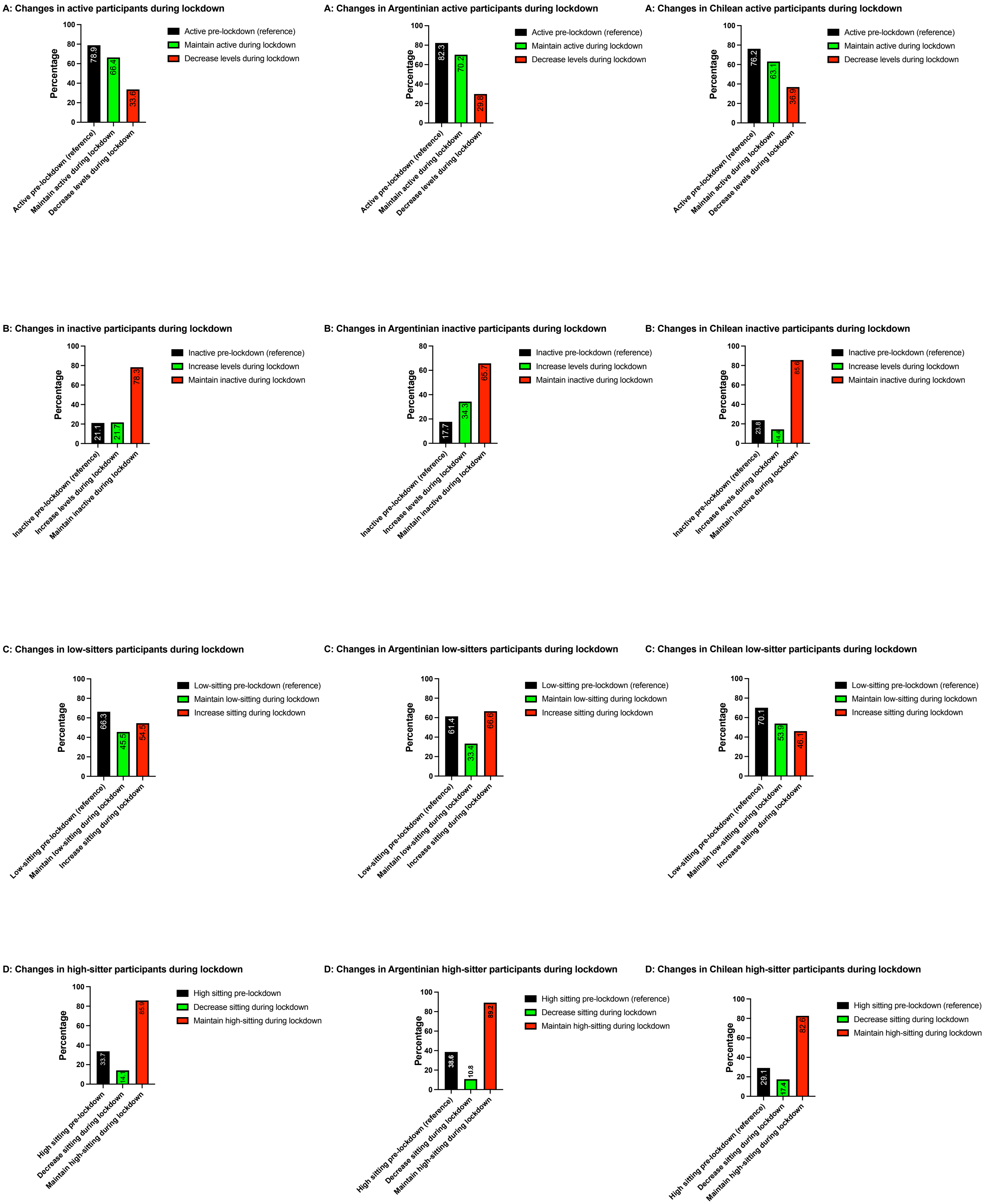

Fig S1: Percentages of the reported changes in physical activity and sitting time **(A)** physical activity changes in subjects who were active before lockdown **(B)** physical activity changes in subjects who were inactive before lockdown **(C)** sitting time changes in subjects who were low-sitters before lockdown **(D)** sitting time changes in subjects who were high-sitters before lockdown.
